# Beyond timing and step counting in 360° turning-in-place assessment: a scoping review

**DOI:** 10.1186/s12938-024-01208-0

**Published:** 2024-01-31

**Authors:** Slavka Netukova, Lucie Horakova, Zoltan Szabo, Radim Krupicka

**Affiliations:** Faculty of Biomedical Engineering, Department of Biomedical Informatics, Czech Technical University, Nam Sitna 3105, Prague, Czech Republic

**Keywords:** Inertial measurement unit, Wearable sensor, Camera system, Balance

## Abstract

**Background:**

Turning in place is a challenging motor task and is used as a brief assessment test of lower limb function and dynamic balance. This review aims to examine how research of instrumented analysis of turning in place is implemented. In addition to reporting the studied population, we covered acquisition systems, turn detection methods, quantitative parameters, and how these parameters are computed.

**Methods:**

Following the development of a rigorous search strategy, the Web of Science and Scopus were systematically searched for studies involving the use of turning-in-place. From the selected articles, the study population, types of instruments used, turn detection method, and how the turning-in-place characteristics were calculated.

**Results:**

Twenty-one papers met the inclusion criteria. The subject groups involved in the reviewed studies included young, middle-aged, and older adults, stroke, multiple sclerosis and Parkinson’s disease patients. Inertial measurement units (16 studies) and motion camera systems (5 studies) were employed for gathering measurement data, force platforms were rarely used (2 studies). Two studies used commercial software for turn detection, six studies referenced previously published algorithms, two studies developed a custom detector, and eight studies did not provide any details about the turn detection method. The most frequently used parameters were mean angular velocity (14 cases, 7 studies), turn duration (13 cases, 13 studies), peak angular velocity (8 cases, 8 studies), jerkiness (6 cases, 5 studies) and freezing-of-gait ratios (5 cases, 5 studies). Angular velocities were derived from sensors placed on the lower back (7 cases, 4 studies), trunk (4 cases, 2 studies), and shank (2 cases, 1 study). The rest (9 cases, 8 studies) did not report sensor placement. Calculation of the freezing-of-gait ratio was based on the acceleration of the lower limbs in all cases. Jerkiness computation employed acceleration in the medio-lateral (4 cases) and antero-posterior (1 case) direction. One study did not reported any details about jerkiness computation.

**Conclusion:**

This review identified the capabilities of turning-in-place assessment in identifying movement differences between the various subject groups. The results, based on data acquired by inertial measurement units across studies, are comparable. A more in-depth analysis of tests developed for gait, which has been adopted in turning-in-place, is needed to examine their validity and accuracy.

## Background

Turning is an essential part of mobility and has a common occurrence in everyday locomotion [1]. It is a complex task which requires the central nervous system to coordinate the body segments reorientation towards a new direction while maintaining dynamic body stability [2].

Turning manoeuvres are altered by age [3] and neurological disorders, such as Parkinson's disease (PD) [4] or stroke [5]. For older adults, about 30% of falls occur during a standing turning movement or while bending [6]. Consequently, falling while turning carries a risk of hip fracture [7, 8]. In PD, turning difficulty is a sensitive predictor of the two key locomotor symptoms: freezing and falling [9]. The high occurrence of turning in everyday life [1] and its association with falls emphasises the importance of research that focuses on turn analysis.

The 360° turn-in-place is a brief assessment test of lower limb function and dynamic balance, requiring individuals to turn in a circle (360 deg.). The 360° turn is indeed a task which demands fine postural control tuning. It is initiated by head rotation, followed by a cranio-caudal rotatory sequence of the trunk and lower extremities [10]. Turning-in-place can be evaluated solely or as part of a balance assessment tool. If appraised solely, the time it takes to complete the turn and/or the number of steps to turn completely around is recorded. It is part of two of the best validated clinical balance scales [11]: the qualitative Performance-Oriented Mobility Assessment (POMA) [12] and the Berg Balance Scale (BBS) [13]. The POMA scores the 360° turn-in-place on a binary scale, whereas BBS rates turning on a 5-point scale based on assistance and/or time needed to complete the turn.

There are other tests used to assess functional status that include turning. The widely utilised Timed Up and Go (TUG) test is one of them [14–16]. To perform the TUG the patient is timed while they rise from an armchair, walk straight three metres, turn and walk back to the chair and sit down again [17, 18]. The performance of turning-in-place as well as the TUG-turn is associated with trunk control [19]. In contrast to turning-in-place, turning which takes place in a TUG, is affected by transitioning between the tasks preceding turning and following turning. Based on the reported results [20–22], it is opined, that task complexity and turn style provide important turn related kinematic differences [22].

Standing turn performance has been used to delineate elderly fallers from non-fallers. Elderly individuals who are at a higher risk of falls take longer than 4 s [13] and six more steps [23] to turn 360°. A time greater than 3.8 s is associated with a significantly increased rate of dependence [24]. The cut-off time of 3.65 s on the dominant side and 3.75 s on the non-dominant best discriminated fallers from non-fallers with multiple sclerosis [25].

PD patients are slower at the turn-in-place and take more steps than healthy controls [9, 26, 27]. Also, the time taken to turn significantly correlated with the number of steps in PD [28]. It has been stated that the mean time to complete a turn in PD is 6 s [29].

In addition to studies focused on fall risk [30], a number of studies used the characteristics of improvement in the 360° turning-in-place to investigate the effects of a targeted exercise programme [31–35]. In older adults the association of a 360 deg. turn-in-place to cognitive domains [36] and self-care ability [37] has been shown. It has been demonstrated that physical performance measured by turning-in-place is one of the strongest predictors of a subsequent driving cessation in older adults [38]. A modified version of the 360° turn—repeated 360° turns, which requires continuous turning for a predefined amount of time, e.g. 1 min, has proven to be effective in provoking freezing of gait (FOG) in PD [39, 40].

The timed 360° turning-in-place test has demonstrated: good intrarater, interrater, and test–retest reliability in stroke [41]; good test–retest reliability in Parkinson’s Disease [29]; excellent intrarater, interrater, and test–retest reliability in PD patients [42]; good intrarater, interrater, and test–retest reliability in people with multiple sclerosis (MS); and good test–retest reliability in older adults [43].

Besides camera-based motion capture systems, which are widely used in laboratory settings [44–46], wearable technologies, especially wearable inertial sensors, have become an important tool in the field of movement analysis. Their advances, such as portability, ease of use, low cost, and low demand for dedicated space [47], makes them suitable for utilisation in a clinical context and opens a promising future for turning analysis outside research laboratories [48]. Instrumented motor tests are nowadays widely accepted [49].

The instrumented turning-in-place task is also increasingly used and gaining importance. Its parameters are derived from inertial sensor measurements which have demonstrated concurrent and construct validity in relation to mobility assessment [50].

Since the instrumented 360° turning-in-place is a relatively recent approach to standing balance analysis, and since the utilised measures can be varied, a scoping review is appropriate to gather available evidence to examine how research is implemented. Therefore, we collected articles on the topic of the instrumented 360° turning-in-place to conduct a scoping review. The objective is to determine the scope of available studies to have an overview of a topic.

### Related works

Several reviews on turning has been published. Chou and Lee [51] briefly reviewed turning deficits in PD, methodological approaches (including 360° turns), and clinical implications. Godi et al. [52] focused on curved walking and turning in older adults and people with PD. Hulbert et al. [53] provided a narrative review of turning deficits in people with Parkinson’s disease. Spildooren et al. reviewed turning problems and freezing of gait in Parkinson’s disease [54]. Manaf et al. [55] reviewed literature on turning ability among stroke survivors.

Although some previous research also included 360 deg. turning-in-place, none of the above-mentioned reviews: (1) focused on instrumented 360 deg. turning-in-place with the aim to review the process of obtaining outcomes, or (2) provided a review across various diseases or disabilities.

## Methods

This current scoping review followed the Joanna Briggs Institute (JBI) recommendations for conducting and reporting scoping reviews [56] and is congruent with the Preferred Reporting Items for Systematic reviews and Meta-Analyses (PRISMA) checklist for scoping reviews (PRISMA-ScR).

### Identifying the research question

The general research question was “What is the current state of evidence regarding the instrumented 360°  turning-in-place?” Following our initial research, three sub-questions were identified:

1. What data acquisition systems are used in turning-in-place analysis?
2. What kinetic, kinematic and/or other measures of physical status beyond the time taken and number of steps are included in the analysis of the instrumented 360°  turning-in-place?
3. Are the results of turning-in-place tasks comparable across studies?

### Identifying relevant studies

#### Eligibility criteria

We included a cross-sectional and cohort of observational studies as well as interventional studies. Only studies utilising an instrumented 360-deg. turning-in-place controlled measurement were included, e.g. studies focusing on at-home monitoring were excluded.

Only papers focusing on instrumented turning-in-place analysis which differentiated between subject groups, brought knowledge about human movement, or examined the hypothesised link between turn performance and pathology were considered. Papers which were written without an intention to elucidate or interpret the results towards new insight into performing a 360° turning-in-place of the subject group, e.g. introducing new parameters or demonstrating novelty algorithms for turn detection, were excluded.

### Article selection

The article selection was performed in two phases: (1) databases search and (2) references traversing.

Searches were performed in the Scopus and the Web of Science databases. The search was performed up to November 2022, with no set beginning. The search strategy included terms relating to the turning-in-place task. The query

(TITLE-ABS-KEY (‘‘standing turn’’) OR TITLE-ABS-KEY (‘‘turn in-place’’) OR TITLE-ABS-KEY (‘‘turning in-place’’) OR TITLE-ABS-KEY (turn-in-place) OR TITLE-ABS-KEY (turning-in-place) OR TITLE-ABS-KEY (‘‘turn in place’’) OR TITLE-ABS-KEY (‘‘turning in place’’) OR TITLE-ABS-KEY (‘‘turn 360’’) OR TITLE-ABS-KEY (‘‘turning 360’’) OR TITLE-ABS-KEY (‘‘360° turning’’) OR TITLE-ABS-KEY (‘‘360° turn’’) OR TITLE-ABS-KEY (‘‘360 deg turning’’) OR TITLE-ABS-KEY (‘‘360 deg turn’’) OR TITLE-ABS-KEY (‘‘rapid turn’’) OR TITLE-ABS-KEY (‘‘standing turns’’) OR TITLE-ABS-KEY (‘‘rapid turns’’) OR TITLE-ABS-KEY (‘‘turns 360’’) OR TITLE-ABS-KEY (‘‘360° turns’’)).

was used for searching in titles, abstracts, and keywords in the Scopus database.

**The query**

‘‘Standing turn’’ (Topic) OR ‘‘turn in-place’’ (Topic) OR ‘‘turning in-place’’ (Topic) OR ‘‘turn-in-place’’ (Topic) OR ‘‘turning-in-place’’ (Topic) OR ‘‘turn in place’’ (Topic) OR ‘‘turning in place’’ (Topic) OR ‘‘turn 360’’ (Topic) OR ‘‘turning 360’’ (Topic) OR ‘‘360° turning’’ (Topic) OR ‘‘360° turn’’ (Topic) OR ‘‘360 deg turning’’ (Topic) OR ‘‘360 deg turn’’ (Topic) OR ‘‘rapid turn’’ (Topic) OR ‘‘standing turns’’ (Topic) OR ‘‘rapid turns’’ (Topic) OR ‘‘turns 360’’ (Topic) OR ‘‘360° turns’’ (Topic).

Was used for searching titles, abstracts, author keywords, and Keywords Plus in the Web of Science database.

No filters were used during searching.

Only research published in the English language was considered and only articles published in peer-reviewed journals (no abstracts and conference papers) were considered.

The search results were analysed by two reviewers (SN and LH). Both reviewers independently selected records suitable for further analysis based on titles and abstracts. Discrepancies between the reviewers were resolved by a third reviewer (RK). Full texts of the articles that met the eligibility criteria were then obtained and reviewed.

As recommended by JBI, a second selection was made through the references of the articles selected in the first phase (SN, LH).

### Charting the data

The conducted research process and results obtained were depicted by a PRISMA flowchart. The distribution over year of the number of included studies was summarised in a chart. For each article information was extracted as follows:

- Publication year,
- Study design,
- Participants' characteristics,
- Turning-in-place measurement conditions,
- Acquisition systems,
- Turn detection methods,
- Kinetic, kinematic and other measures,
- Custom methods of measures computation,
- Objectives and
- The main results related to turning-in-place obtained.

## Results

### Article selection

**Fig. 1:**
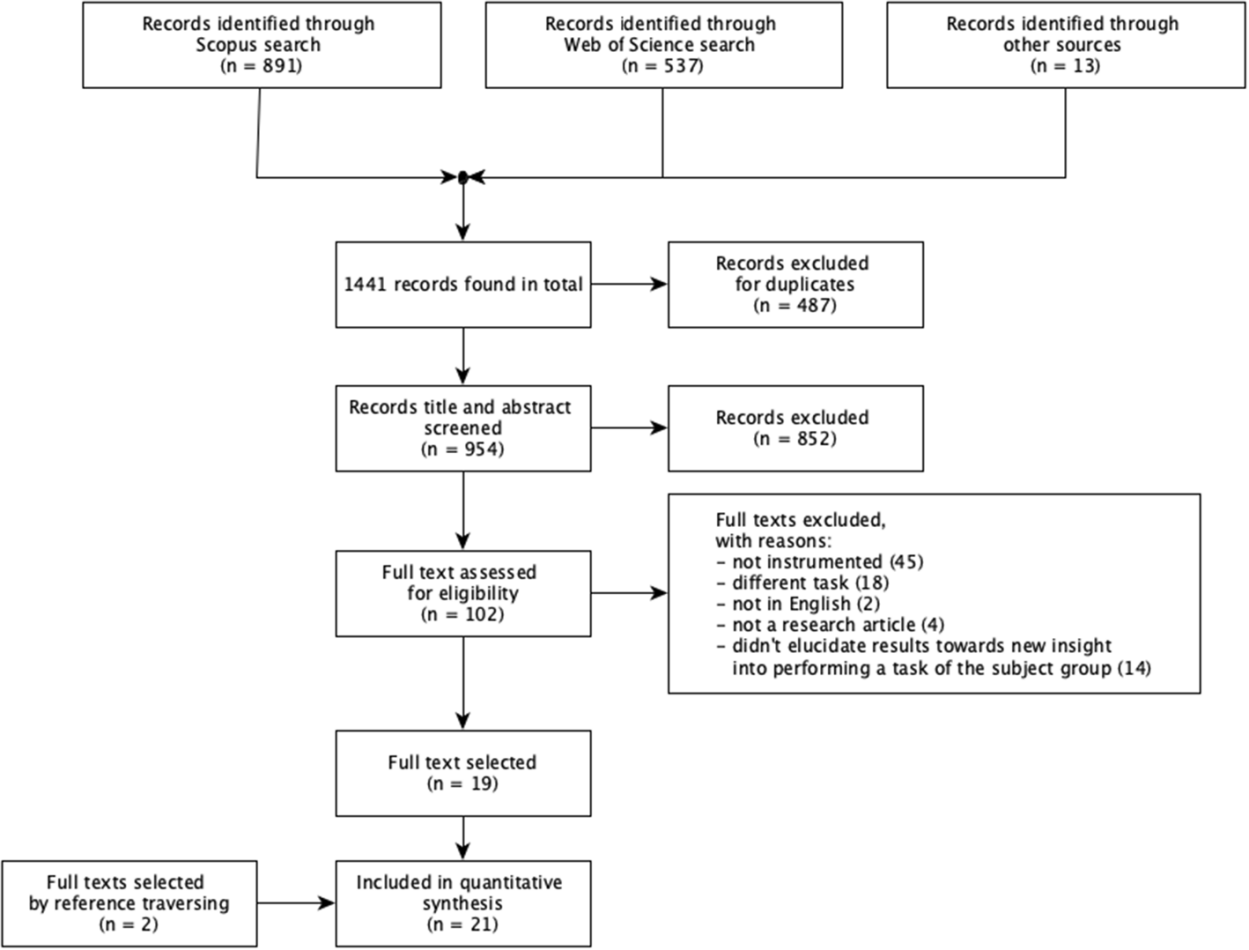
Paper selection flowchart

The PRISMA flowchart (Fig. 1) shows the results for each selection phase. In the first phase, the literature search identified a total of 1441 articles on Scopus, Web of Science, and other sources. After the primary selection, 19 articles met eligibility criteria and were included in the synthesis. In the second phase, 2 additional articles were retrieved by traversing through the synthesis references included in the articles.

### Study characteristics

#### Publication year

**Fig. 2:**
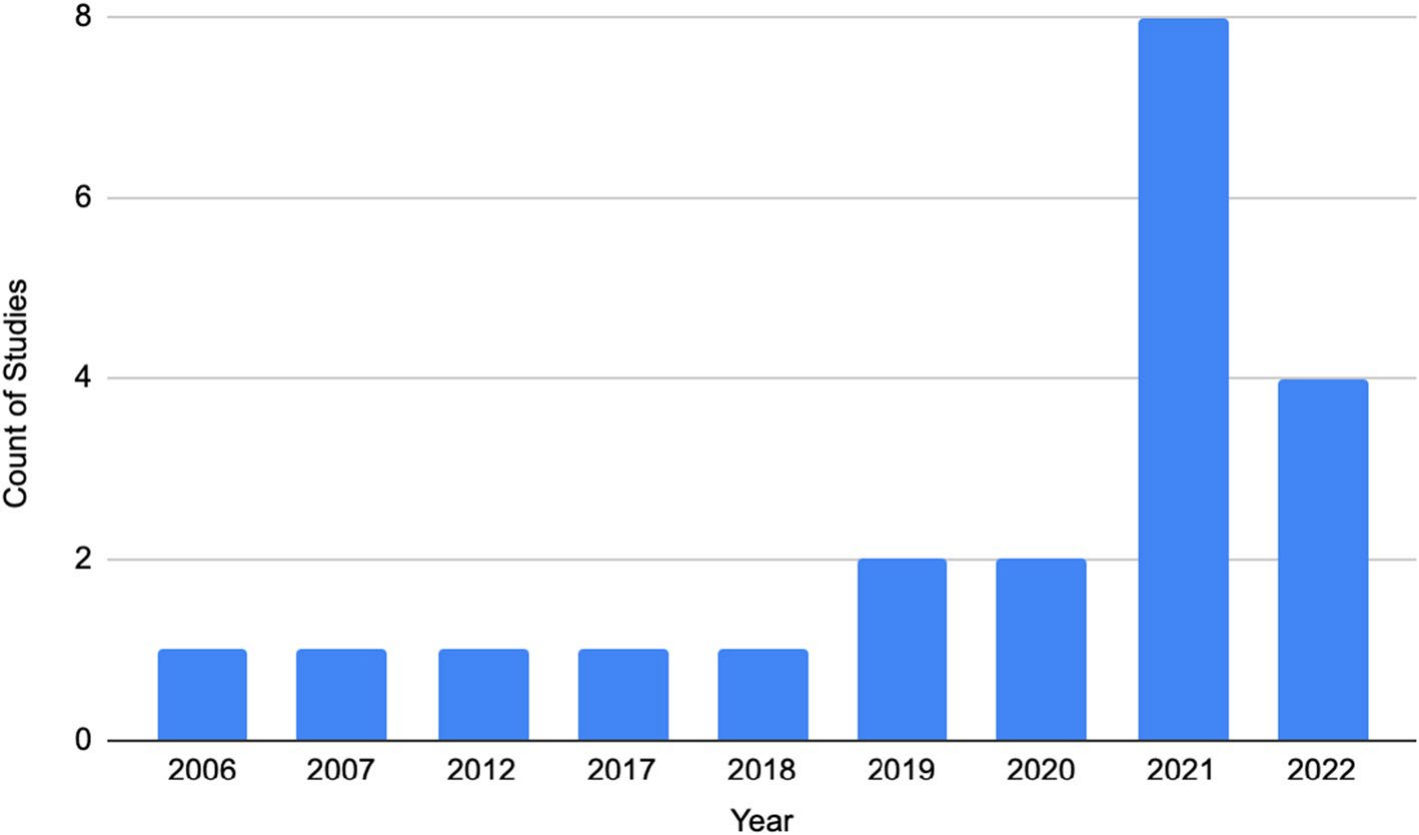
Publication activity over the years

The earliest publication that met our criteria was from the year 2006. The year that had the most articles was from 2021 (Fig. 2).

#### Design

Five [57–59, 62, 77] of the included studies were interventional studies. The other studies were observational. From a total count of 16 observational studies, one study [76] was a prospective study. There were no cohort studies.

### Studied populations

**Fig. 3:**
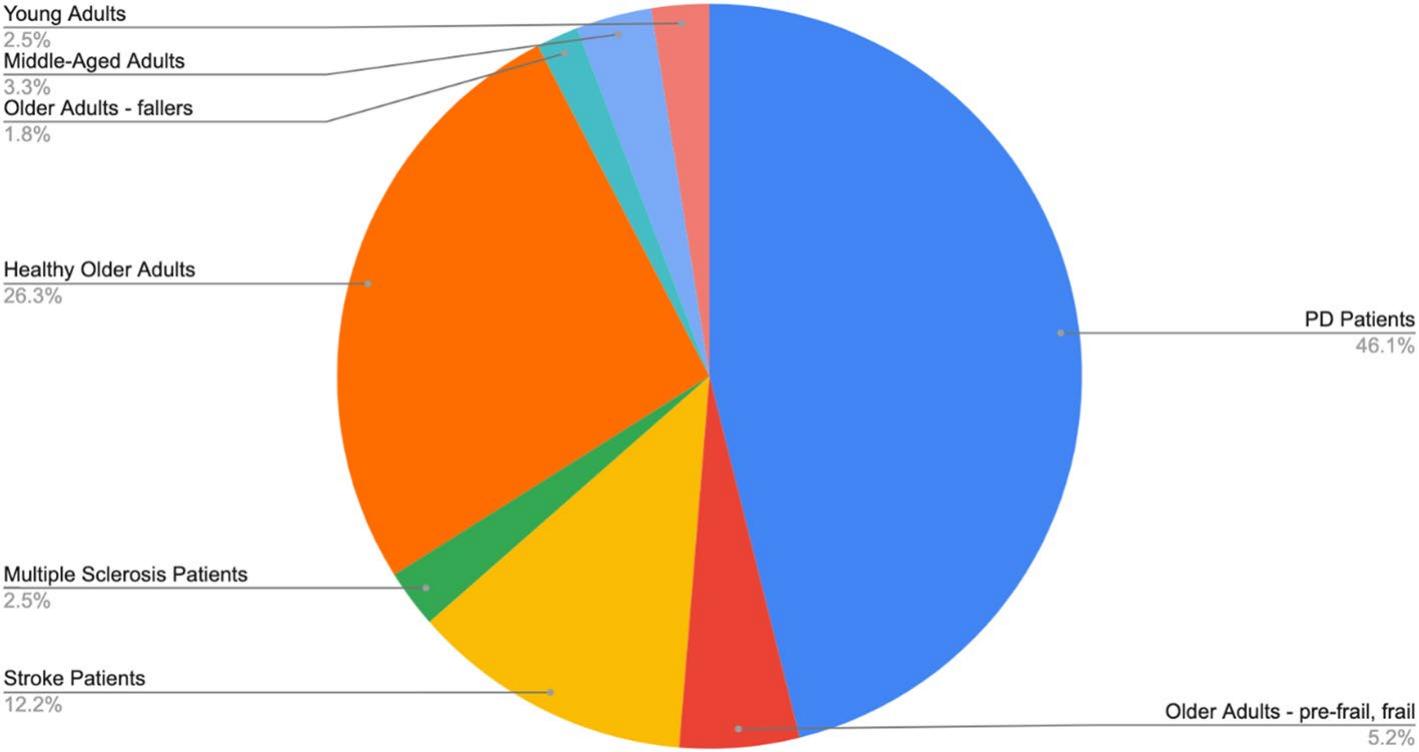
Composition of subject groups in the reviewed articles

Six studies out of 21 investigated solely one subjects' group [57, 59, 64, 69, 70, 77]. Other studies included two [58, 61, 62, 66, 71, 72] or three subjects’ groups [60, 63, 65, 67, 68, 73–76]. Included groups were PD patients [57–68], stroke patients [69–72], people with multiple sclerosis [75], older adults [58, 60, 62, 63, 65, 67, 68, 71–76], frail and pre-frail older adult [73], older adult fallers [76], middle-aged adults [74, 75], and young adults [74, 77] (Table 1). In total, the studied population amounted to 1193 subjects (664 males, 510 females). The distribution of subject groups is depicted on Fig. 3.

**Table 1:** Population information of the included studies

Item	Variables	Number of studies	Studies
Population	PD patients (excluding PD freezers and PD non-freezers)	3/21	[57–59]
	PD freezers	9/21	[60–68]
	PD non-freezers	7/21	[60, 61, 63, 65–68]
	Stroke patients	4/21	[69–72]
	Healthy older adults	13/21	[58, 60, 62, 63, 65, 67, 68, 71–76]
	Middle-aged adults	2/21	[74, 75]
	Young adults	2/21	[74, 77]
	People with multiple sclerosis	1/21	[75]
	Frail older adults	1/21	[73]
	Pre-frail older adults	1/21	[73]
	Older adults—multiple fallers	1/21	[76]
	Older adults—single-fallers	1/21	[76]
Number of subjects	< 20	2/21	[59, 77]
	20–40	7/21	[57, 58, 64, 70–72, 76]
	40–60	5/21	[60, 61, 63, 65, 66]
	60–100	5/21	[62, 67, 69, 74, 75]
	> 100	2/21	[68, 73]

### Testing conditions

Two types of turns were used: turning limited by the number of turns and turns limited by total time. All studies (i.e. 13 studies) involving turning limited by the number of turns utilised a single turn in the turning task [58, 59, 65, 67, 69–77]. Eight studies limited turning by the total time of 1 min [61–63, 66, 68], 2 min [60, 64], or 80 s [57]. Two out of these five studies started their measurement with 20 s of quiet standing [57, 60].

The majority of studies included both left and right turns for measurement [57–71, 73–75, 77]. Time limited turning-in-place alternated turns to the right and left.

Only four studies included instructions to turn quickly [58, 62, 64, 66]. Other studies instructed participants to turn at a self-selected speed of turn [57, 60, 63, 67, 69–72, 76, 77].

or did not report instructions given for turn speed [59, 61, 65, 68, 73–75].

One study employed a self-initiated start [76], one employed a start on command [60]. The rest of the studies did not report any kind of initiation of measurement.

Turning under the dual task condition was studied by STROOP [62], auditory Modified AX-Continuous Performance Task [57, 60], and serial subtractions by 3 s [61, 66].

### Data acquisition systems

Out of all of the studies, 16 acquired data via inertial measurement units [57, 59–66, 68–70, 72–75], three solely via camera motion system, and two employed camera motion system along with force platforms [71, 76].

**Fig. 4:**
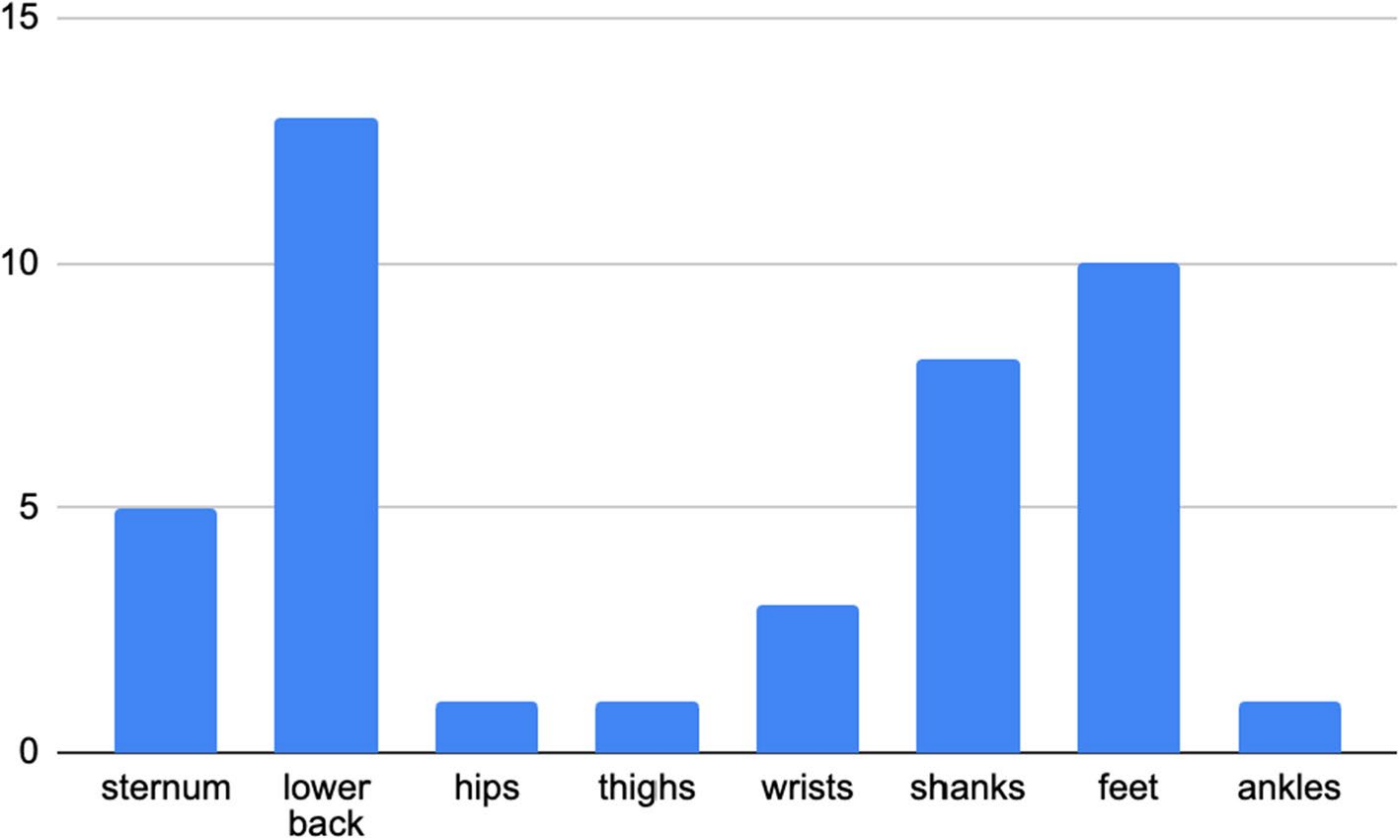
Frequency of sensor placement on body parts

Apart from three studies [64, 65, 68], all studies employing IMU placed one of the sensors on the lower back, Fig. 4. Regarding camera systems, whole body motion data were collected via Human Body Model 2 [78] in one study [71] and via Plug-In Gait marker set [79] also in two studies[67, 76]. A custom marker set was used in two studies [58, 77].

### Signal filtering

Out of 21 studies, 13 studies did not report the approach used to filter the measured signal [57–59, 62, 63, 65, 66, 68, 73–77]. All other studies used a low pass Butterworth filter signal with cut-off frequencies 1 Hz [61], 1.5 Hz [60, 69, 70], 5 Hz [72], and 30 Hz [64] for angular velocity, and 6 Hz [67] for marker trajectory. One study did not report cut-off frequency [71]. Out of the 8 studies reporting utilisation of the Butterworth filter, three stated the order of the filter [67, 71, 72].

### Turn detection methods

#### IMU-based measurement

Two studies referenced commercially available software used for detection [74, 75].

For custom-designed algorithms, the sensors placed near the body centre of mass were used [60–62, 69, 70]. Recorded angular velocity about the vertical axis [60–62] and magnetometer signals [61] were employed to isolate the turn.

Three studies [60, 69, 70] referenced research presented by El-Gohary et al. [80], two studies [69, 70] referenced research presented by Pearson et al. [81], and one study [57] referenced research presented by Mancini et al. [40]. Two studies used changes in angular velocity direction to determine the start and finish of each turn [61, 62].

Details on processing the turning-in-place task are neither described nor referenced in eight articles [59, 63–66, 68, 72, 73].

#### Camera-based measurement

Four different custom approaches were used to detect the turn beginning and end. The first utilised the point where the rotational velocity of the first of the three measured body segments crossed zero to identify the beginning of the turn, and the point when the rotational velocity of the last of those segments returned to zero to identify end of the turn [76]. The second reported foot contact and foot off identification and verification as visually carried out using the digital video recordings and force plates [71]. The third method measured the trunk angle as the angle between a line drawn from one acromion to the other and a line drawn from one floor marker to another [58, 77]. The fourth approach detected the start and end of the turn from the pelvis rotation angle [67].

### Employed quantitative assessment

#### One 360° turn-in-place

Eight studies dealing with one 360° turn-in-place were conducted with an IMU and five with camera systems. The types of kinematic variable were varied, including mainly mean angular velocity [69, 70, 72, 73], peak angular velocity [74, 75], and mean angle [74, 75] (Table 2). The only three other parameters that were used to quantify turning was turn duration [70, 73–75], number of turn cycles [71, 72], and the number of steps taken to complete the turn [58, 65].

**Table 2:** Kinematic and other variables, and source signals used in one 360° turn-in-place assessment

	Overall data acquisition systems	Variable	Source data for variable		
			Sensor location	Signal	Axis/plane
[73]	3 IMU sensors	Mean angular velocity	NR—computed by proprietary software of the sensors manufacturer		
		Turn duration	NR—computed by proprietary software of the sensors manufacturer		
[69]	3 IMU sensors	Mean angular velocity	lower back	Angular velocity	Yaw
[70]	3 IMU sensors	Mean angular velocity	NR—computed by proprietary software of the sensors manufacturer		
		Turn duration	NR—computed by proprietary software of the sensors manufacturer		
[75]	3 IMU sensors	Peak angular velocity	NR	NR	NR
		Mean angle	NR—computed by proprietary software of the sensors manufacturer		
		Turn duration	NR—computed by proprietary software of the sensors manufacturer		
[74]	3 IMU sensors	Peak angular velocity	NR	NR	NR
		Mean angle	NR—computed by proprietary software of the sensors manufacturer		
		Turn duration	NR—computed by proprietary software of the sensors manufacturer		
[65]	2 Gyroscopes	Number of steps	Shank	Angular velocity	Pitch
		Turn duration	Shank	Angular velocity	Pitch
[59]	9 IMU sensors	Turn velocity	NR	NR	NR
		Turn duration	NR	NR	NR
[72]	4 IMU sensors	Mean angular velocity	Trunk	Angular velocity	Pitch
		Mean angular velocity	Lower back	Angular velocity	Pitch
		Mean angular velocity	Shank	Angular velocity	Pitch
		Mean angular velocity	Trunk	Angular velocity	Yaw
		Mean angular velocity	Lower back	Angular velocity	Yaw
		Mean angular velocity	Shank	Angular velocity	Yaw
		Mean angular velocity	Trunk	Angular velocity	Roll
		Mean angular velocity	Lower back	Angular velocity	Roll
		Range of motion	Trunk	Angular velocity	Pitch
		Range of motion	Lower Back	Angular velocity	Pitch
		Range of motion	Shank	Angular velocity	Pitch
		Range of motion	Lower Back	Angular velocity	Roll
		Range of motion	Trunk	Angular velocity	Pitch
		Turn duration	Shank	Angular velocity	Yaw
		Number of cycles	Shank	Angular velocity	Pitch
[76]	Motion camera system, force plate	Peak/mean head–trunk angle	Head, trunk	Position	NR
		Peak/mean trunk–pelvis angle	Trunk, pelvis	Position	NR
		Turn duration	Head, trunk, pelvis	Position	TP
		Number of steps	NA	Force + NR	NA
		Trunk onset	Trunk	Position	NR
		Pelvis onset	Pelvis	Position	NR
		Foot onset	NR	NR	NR
		CoP to head time	Head	Force + position	NR
		CoP to CoM time	Whole body	Force + position	NR
[71]	Motion camera system, force plate	Total/mean double support time (s)	NR	Force + position	NR
		Total/mean critical time	NR	Force + position	NR
		Number of turn cycles	NR	NR	NR
[77]	Motion camera system	Turn angle	Trunk	Position	TP
		Turn velocity	Trunk	Position	TP
[58]	Motion camera system	Turn angle	Trunk	Position	TP
		Number of steps	NR	NR	NR
		Turn duration	NR	NR	NR
[67]	Motion camera system	Turning area	NR	Position	NR
		AP-RMS/ML-RMS distance	NR	Position	TP
		Number of steps	NR	NR	NR
		Turn duration	NR	NR	NR

*NR* not reported, *NA* not applicable, *AP* antero-posterior, *ML* medio-lateral, *CoP* centre of pressure, *CoM* centre of mass, *TP* transverse plane

The most frequently evaluated parameters on camera system utilised in the studies were turn duration [58, 67, 76], number of steps [58, 67, 76], and turn angle [58, 77].

One study used a camera system and analysed seven additional turning variables: head onset to trunk onset time, trunk onset to pelvis onset time, pelvis onset to foot off time, peak head–trunk angle, peak trunk–pelvis angle, mean head–trunk angle, mean trunk–pelvis angle (all angles in the transverse plane), COP onset to head onset time and COP onset to COM onset time [76]. In one study based on turn segmentation the following phases were identified: single support, double support with feet apart, and double support with feet together, and their corresponding time intervals were defined. This way, 14 time and 4 other parameters were assessed: the mean values of seven time parameters (leading limb critical times, trailing limb critical times, critical time duration, double support feet apart, double support feet together, double support duration, time taken in a turn cycle), their standard deviations and ratios (number of critical time/single support), total times (total critical time duration, total double support duration), and number of turn cycles [71]. One study calculated the turning area and RMS from the CoM position [67].

#### Time limited 360° turning-in-place

All studies (i.e. eight studies) dealing with a time limit for the 360-deg. turning-in-place employed an IMU for data acquisition and, with exception of two articles ([64, 68]), they all used at least peak angular velocity to quantify turning. Two studies also used mean angular velocity and one study used range of acceleration (Table 3). Other variables are dominantly derived from acceleration signals: jerkiness [60–63, 66], freezing-of-gait ratio [60, 63, 64, 66, 68], turn duration [57, 60, 61], number of steps [61], and number of turns [66].

**Table 3:** Kinematic and other variables, and source signals used in time limited 360° turning-in-place assessment

	Overall data acquisition systems	Variable	Source data for variable		
			Sensor location	Signal	Axis
[60]	8 IMU sensors	Peak angular velocity	Lower back	Angular velocity	Yaw
		FOG Ratio	Shank	Acceleration	AP
		Jerkiness	NR	Acceleration	ML
		Number of turns	Lower back	Angular velocity	Yaw
		Turn duration	Lower back	Angular velocity	Yaw
[61]	3 IMU sensors	Peak angular velocity	NR	Angular velocity	Yaw
		AP range of acceleration	NR	Acceleration	AP
		ML range of acceleration	NR	Acceleration	ML
		Turn duration	Lower back	Magnetometer signal	Planar
		Number of steps	feet	Angular velocity	Pitch
		AP jerkiness	NR	Acceleration	AP
		ML jerkiness	NR	Acceleration	ML
[62]	1 IMU sensor	Mean/peak angular velocity	Lower back	Angular velocity	Yaw
		Jerkiness	Lower back	Acceleration	ML
[63]	3 IMU sensors	Mean/peak angular velocity	NR	NR	NR
		Jerkiness	NR	Acceleration	ML
		FOG ratio	Shank	Acceleration	AP
[57]	8 IMU sensors	Peak angular velocity	NR	NR	NR
		Turn duration	NR	NR	NR
[64]	IMU sensors^a^	FOG ratio	Shank	Acceleration	AP
[66]	8 IMU sensors	Peak angular velocity	Trunk	Angular velocity	Yaw
		FOG ratio	Shank	Acceleration	AP
		Jerkiness	NR	NR	NR
		Number of turns	Trunk	Angular velocity	Yaw
[68]	IMU sensors^a^	FOG ratio	Ankle	Acceleration	AP

^a^Total number of sensors is not reported, *NR* not reported, *AP* antero-posterior, *ML* medio-lateral

### Custom computation of quantitative measures

A portion of quantitative measures is custom implemented. From these measures, we extracted measures which were nontrivial and were employed in two or more studies.

For IMU measurements, parameters which were nontrivial and were calculated by custom implementation included the FOG ratio, jerkiness, and the number of steps.

In all cases the FOG ratio was calculated from antero-posterior shank or ankles acceleration signals [60, 63, 64, 66, 68], according to the formula suggested by Mancini et al. [66]. No study publicised whether the source signal came from the inner or outer leg.

Jerkiness used to quantify fluidity of turning was calculated as the integral of the squared time derivative of the linear acceleration (integrated squared jerk) in the medio-lateral direction [60–63] and the antero-posterior direction [61]; one paper did not report a method or source signal for jerk estimation [66].

Step detection required for counting steps was based on peak detection in the medio-lateral component of the angular velocity [61, 72] and a continuous wavelet analysis of angular velocity around the medio-lateral axis [65].

For camera-based measurements, there are nontrivial custom implemented measures, but none of them appeared in at least two studies.

### Studies objectives

The majority of studies (12 studies) investigated turning-in-place in relation to PD. It included an investigation on the association between pre-frontal cortex activity and turning [57, 60], the effect of physical training on turning performance in PD patients experiencing freezing of gait (FOG) [62], the influence of dopaminergic medication on dynamic balance control [63], differences between turning while walking and turning-in-place for patients with and without FOG [61], determination whether individuals with PD can use visual, vestibular, and proprioceptive cues to estimate how far they have turned [58], the association between different domains of postural control and the severity of FOG [64], the association between cognitive performance and FOG severity [68], the contribution of spatial constraints on turning in patients with and without FOG [65], the resistance to high levodopa doses of FOG [59], the immediate effectiveness of open- and closed-loop cueing in improving turning characteristics [66], and analyse the walking and turning characteristics in order to define the characteristics of FOG [67].

Studies targeting stroke patients (4 studies) analysed the association between turning mobility and cognitive function (via Mini-Mental State Examination) [69], explored the relationship between turning performance and trunk function [70], and characterised differences in turning kinematics between patients and older adults [71, 72].

Other studies aiming to discriminate subjects groups via turning-in-place quantification include differentiation between older adults with frailty from those without [73], neurotypical young adults, middle-aged adults, and older adults [74], older community-dwelling multiple fallers and non-fallers [76], neurotypical older adults and middle-aged people with multiple sclerosis [75], and middle-aged neurotypical adults and people with multiple sclerosis [75].

The purpose of one study was to determine whether walking on a rotating disc would cause changes in the perception of turning in young adults [77].

### Studies key results

#### Differentiation by quantitative measures of turning

In PD patients, the mean turning velocity differentiated PD freezers and PD non-freezers from older adults, but not PD freezers from PD non-freezers [63]. The PD group showed a difference in step length, in the asymmetry index of step length at turning, but not in step time and the asymmetry index of step time compared to controls [67].

A significant group-medication effect for the FOG ratio was found in PD freezers and non-freezers, whereas the mean turning velocity, peak turning velocity and turn jerkiness were not significantly affected by medication in these groups [63]. Results revealed no significant difference between PD medication for turning performance (turn peak velocity, turn duration) [57]. For dual task turning outcomes in PD with FOG, significant interaction effects between treadmill training (tied or split belt) and the time of measurement (pre-, post- training and retention) were found for mean and peak turning velocity [62]. A significant interaction in the group-turn in PD freezers and non-freezers for the turn duration and number of steps was demonstrated, but not for peak velocity, acceleration range, and jerkiness [61]. The group effect was exhibited for turn duration, number of steps, and peak velocity [61].

The mixed model detected an interaction between groups (PD freezers, PD non-freezers, controls) and the area dedicated to turning for step count and turn duration [65].

As for intervention, doubling the levodopa infusion rate in PD significantly improved both tested turn parameters: duration and velocity [59]. Relating to cueing, the freezing ratio and the jerkiness significantly decreased with cueing compared to the baseline in both freezers and non-freezers [66]. In addition, freezers and non-freezers benefited from cueing to a different degree [66]. Another analysis showed a different number of steps taken for the PD group compared to the controls, but not in the time taken to execute turns after cueing [58]. Subjects with PD were just as accurate as controls when turning with their eyes open or eyes closed—there was no statistically significant difference in the turning angle between the groups [58].

Post-stroke patients took a significantly longer time to turn [72] more turn cycles than healthy individuals [71, 72]. They also had a more critical time and a higher number of single support critical phases than healthy counterparts [71]. Also, the post-stroke group showed lower values angular velocity for sternum, sacrum, and shank in flexion–extension [72], and higher values for range of motion for the sternum and sacrum in lateral bending and flexion–extension [72].

Regarding other subject groups, turn angle-based parameters have been able to distinguish young adults from middle-aged adults [74], middle-aged adults from older adults [74], and middle-aged adults from pwMS [75]; turn duration-based parameters distinguished non-frail older adults from frail [73], pre-frail older adults from frail [73], middle-aged adults from older adults [74], and middle-aged adults from pwMS [75]; turn velocity-based parameters have distinguished non-frail older adults from frail [73], pre-frail older adults from frail [73], young adults from middle-aged adults [74], middle-aged adults from older adults [74], middle-aged adults from pwMS [75]. Measurements of segmental orientation when turning revealed differences between non-fallers and multiple fallers from pelvis onset time, mean head–trunk angle, peak trunk–pelvis angle, mean trunk–pelvis angle [76]. For details see Table 4.

**Table 4:** Turning measures and their ability to differentiate between subject groups

	Differ	Does not differ
Turn angle	Middle-aged adults vs. older adults* [74]	Young adults vs. Middle-aged adults* [74]; older adults vs. pwMS* [75]; PD patients vs. controls [58]
Turn angle variability*	Young adults vs. middle-aged adults [74]; middle-aged adults vs. older adults [74]; middle-aged adults vs. pwMS [75];	–
Turn duration	Non-frail vs. frail [73]; pre-frail vs. frail [73]; middle-aged adults vs. older adults* [74]; middle-aged adults vs. pwMS*[75]; post-stroke patients vs. controls[72]	Multiple fallers vs. non-fallers [76]; young adults vs. middle-aged adults* [74]; PD patients vs. controls [58]
Turn duration variability*	Middle-aged adults vs. pwMS [75];	Young adults vs. middle-aged adults [74]; middle-aged adults vs. older adults [74];
Mean turn velocity	Non-frail vs. frail [73]; pre-frail vs. frail [73]; older adults vs. PD non-freezers [63]; older adults vs. PD freezers [63]; post-stroke patients vs. controls [72]	PD freezers vs. PD non-freezers [63];
Peak turn velocity	Older adults vs. PD non-freezers [63]; middle-aged adults vs. older adults* [74]; middle-aged adults vs. pwMS* [75]; older adults vs. PD freezers [63];	PD freezers vs. PD non-freezers [63]; young adults vs. middle-aged adults* [74]; older adults vs. pwMS* [75];
Peak turn velocity variability*	Young adults vs. middle-aged adults [74]; middle-aged adults vs. pwMS [75];	Middle-aged adults vs. older adults [74]
FOG ratio	Older adults vs. PD freezers [63]; PD freezers vs. PD non-freezers [63];	–
Jerkiness	Older adults vs. PD freezers [63]; PD freezers vs. PD non-freezers [63];	–
Pelvis onset time	Multiple fallers vs. non-fallers [76]	–
Mean head–trunk angle	Multiple fallers vs. non-fallers [76]	–
Peak trunk–pelvis angle	Multiple fallers vs. non-fallers [76]	–
Mean trunk–pelvis angle	Multiple fallers vs. non-fallers [76]	–
Number of steps	PD patients vs. controls [58]	–
Number of cycles	Post-stroke patients vs. controls [71, 72]	–
Mean angular velocity in flexion–extension	Post-stroke patients vs. controls [72]	–
Mean angular velocity in lateral bending	Post-stroke patients vs. controls [72]	–

*pwMS* people with multiple sclerosis

^*^Calculated from multiple measurements for the subject

Regarding intervention by passive stimulation on a rotating disc, it was demonstrated that following stimulation, subjects consistently and significantly overshot their targets (i.e. exceeded the turn amplitude) in active trials when asked to turn in the direction opposite the disc rotation [77].

#### Association of quantitative measures of turning

In PD patients, an association between turning performance with pre-frontal cortex activity of non-freezers was demonstrated for only the number of turns completed in the dual-task condition, whereas in freezers a higher pre-frontal activity was associated only with the FOG ratio in the single-task condition [60].

The MDS-UPDRS III correlated with turn duration and the number of steps for PD patients without freezing, while it did not correlate to any measure in PD patients with freezing [61]. The Posture Instability and Gait Disability (PIGD) subscore correlated with all the turning measures in PD without freezing, specifically, with turn duration, number of steps, turn peak velocity, jerkiness, and range of acceleration [61]. Instead, the PIGD subscore was significantly associated with turn duration only in the PD with freezing [61]. Neither disease duration nor MoCA was associated with any turning measure [61].

Regarding the association of FOG severity measured as FOG ratio to postural control, medio-lateral CoP amplitude in quiet standing, there was a significant correlation, explaining 30% of the FOG ratio variance [64]. There were associations between the FOG ratio and cognitive performance in either PD freezers or non-freezers [68]. The severity of FoG correlated with the total step count, total step time, and walking speed during turning [67].

In patients with a stroke, the mean angular velocity of the paretic side was significantly associated with the MMSE score whereas the non-paretic side was not [69]. When turning toward the paretic side, angular velocity correlated with trunk flexibility [70]. Turn duration is negatively correlated with trunk muscle strength and trunk impairment scale [70]. No association of turn duration and angular velocity was found when turning toward the non-paretic side [70].

## Discussion

### Summary of evidence

This scoping review was aimed at examining current research regarding turning-in-place, its measurement conditions, performance measures and methods of computation.

The turning conditions were mainly consistent across the studies. Two different forms of turning-in-place were found: time limited turning and a one timed turn. Usually, participants were instructed on the turning speed. Only two studies reported giving instructions to start the turning task. However, there is evidence that gait initiation is influenced by disease, e.g. Parkinson disease [82], gait ignition failure syndrome [83], Huntington’s disease [84]. Therefore, whether the turn started on command or self-initiated might play an important role on the outcome.

All studies included both males and females in the analysis. However, the proportion of representatives in the groups was not balanced. Based on knowledge from previously published differences between genders in gait performance [85, 86], the question arises whether gender-related differences occur in turning.

### Used acquisition systems

The quality and validity of movement analysis are dependent on the measurement instruments used [87]. However, there is no defined consensus on what parameters should be computed when using different data acquisition systems, e.g. camera system, inertial units. Movement parameters are influenced by data processing techniques [88]. Comparisons should be performed across systems to determine which systems are appropriate with respect to specific parameters. Regarding systems producing continuous signals, there is a concern for the reliability of gait curves obtained when movement tasks are performed repeatedly. In most cases, the curves are very similar for one subject. However, curves are scattered occasionally and a selection of curves characterising the subject must be accomplished [89]. Therefore, the reliability of signals obtained should be investigated and the interpretation of results should be careful to identify how much the parameters' values are driven by movement and how much was due to the function of data acquisition and processing techniques.

The feasibility of inertial measurement units to quantify body kinematics has been demonstrated [90] and their utilisation in movement analysis has increased [91]. Thus, their employment in most of the studies is not surprising. On the other hand, camera systems take advantage of markers to capture the precise position of body segments and make their accurate mutual position, angles, or rotation available. A single use of force platforms was exhibited. Besides counting the number of steps required to turn, force platform data can assist in obtaining parameters derived from the centre of gravity [92], ground reaction force [93], lower-limb joint moments or power [94].

### Employed measures

If we focus on the parameters utilised in turning-in-place assessment, the most frequently used spatio-temporal parameters were mean [69, 70, 73–75] and peak [74, 75] values of the measured signals. This is probably because the calculation is simple, and the interpretation is intuitive.

Most data acquisition systems provide continuous signals in three dimensions over the whole turn and these signals can be useful data sources. Thus, new more complex parameters should be proposed to improve quantitative turning-in-place assessment. In comparison to single parameter analysis, analysis of the continuous curve is more informative [95]. New parameters might embody the shape of the turn curve and could provide movement related interpretation.

A few studies did not specify the method of the parameter’s calculation [59, 63, 66, 70, 73–75]. Many of those studies used commercial data acquisition systems including software for movement parameters reporting. Which is why it can be expected that the parameters were calculated by the software. In that case, the parameters are comparable across studies. On the other hand, it should be mentioned that the calculation is usually black-box and it cannot be guaranteed that the calculation method does not differ between software versions. Thus, results reported by new software versions should be validated first.

Regarding smoothness of movement, all studies estimated jerkiness from linear acceleration. However, there is still neither analysis nor consensus on the most appropriate measure to use in different tasks or with different measurement technologies. Movement smoothness is highly task dependent [96]. Jerkiness derived from IMU acceleration are sensitive to different amounts of orientation reconstruction errors and jerkiness calculation from acceleration data without an estimate of IMU orientation should be avoided [97]. As turning-in-place is basically a rotational movement, it seems to be natural to use gyroscope data. In such a case, SPARC can be applied to gyroscope data without any modifications to jerkiness estimation [97]. Therefore, additional effort could be paid to the selection of the jerkiness indicator for turning-in-place.

Only a few studies performed automatic step counting via measured signal analysis. In all these cases, the step detection algorithm was adopted from the algorithm for detecting steps in walking. People with advanced PD typically experience an altered gait patterns, making it difficult to identify and reliably detect gait events or calculate gait parameters compared to healthy individuals [98]. Though, to our best knowledge, none of the algorithms used was validated for turning-in-place movement.

Most papers included at least one analysed group of PD patients. Reasonably, they used the FoG ratio and consistently employed lower limbs' signal to its computation. Turning-in-place is critical since it is a quick, easy test to perform and suited for quick clinical evaluation [71]. The need to consider turning manoeuvres in routine clinical practice has been suggested [1]. Single inertial measurement units have shown their capability to be used in clinical environments to aid diagnosis and severity assessment, determine rehabilitation and intervention efficacy, and delineate pathological groups from healthy controls [99]. Based on the previous statements, a reduction in the number of sensors can be expected to simplify and accelerate assessment. Therefore, the development of the FoG ratio’s counterpart from chest or lumbar sensors may prove to be useful.

Taking advantage of time limited turning-in-place, i.e. a few repetitions of the same task, changes in movement rhythm can be evaluated. For example, a recent pioneering study showed promising results achieved using a single parameter, which combined both amplitude decrement and decrease in movement velocity in the repetitive finger tapping task in patients with neurological disorder [100].

Based on the evidence, walking-turn derived parameters are less reliable than gait parameters for straight locomotion [101, 102]. With regard to gait research, lower reliability, i.e. higher variability, could initiate an investigation [103]. Like gait, the analysis of turning-in-place variability may gain new knowledge relating to specific types of pathology.

It would be beneficial for researchers if a range of “normal” values was defined for both discrete indicators and continuous signals, such as movement patterns (curves). In this way, new subjects can be classified as belonging or not belonging to the group [104]. It has been shown that prediction bands are an adequate statistical tool to apply to continuous data of gait [89, 104–106], walking turns [107], sit-to-stand movement [108], cervical spine movement [109], and scapulo-humeral coordination [110]. Despite growing interest in turning-in-place analysis, there currently is no definition of normal ranges.

### Comparability of studies

This review examined how research is conducted in the field of turning-in-place. Consistency in data collection and processing is essential for comparing the results of different studies. Considering the main previous outlines, i.e.: (1) only two turning-in-place variants (time limited turning, one turn); (2) significant predominance of one type of acquisition system (inertial measurement units); and (3) low diversity in output measures and their computation, reviewed studies are suitable for comparison.

### Summary

Finally, low variability in turning conditions, measurement approaches, and low heterogeneity in the employed indicators suggest well-formed opinions can be constructed from the collected results. Considering the higher number of reviewed studies including PD patients and older adults, results of this scoping review suggest the opportunity for a systematic review and possibly meta-analyses.

## Conclusion

Instrumented turning-in-place has proven to be a task capable of differentiating between subject groups. It has been shown that the subjects’ performance of this task is related to muscle strength, cognitive performance, and other measures.

To meet the aim of this scoping review which is determining the scope of available studies using of instrumented turning-in-place task, we identified 20 articles on this topic. Two types of turning were identified: (1) one turn and (2) time-limited turning. The main portion of studies focused on Parkinson’s disease patients’ motion and utilised inertial measurement units. Besides dominating descriptive measures of kinematic signals, such as mean and peak value, turn duration, jerkiness and FoG ratio were also evaluated multiple times.

We have made subtle suggestions in turning-in-place data processing, but we also understand the importance of the employed parameters and their close relationship to the subject groups. The aim of our suggestions is to point out provided courses for future research, but not to cover all reviewed subject groups and provide deep insight into related movement distinctions.

## Data Availability

The data that support the findings of this study are available from the corresponding author upon reasonable request.
